# The dialogue between protozoa and bacteria in a microfluidic device

**DOI:** 10.1371/journal.pone.0222484

**Published:** 2019-10-09

**Authors:** Anna Gaines, Miranda Ludovice, Jie Xu, Marc Zanghi, Richard J. Meinersmann, Mark Berrang, Wayne Daley, Doug Britton

**Affiliations:** 1 Aerospace, Transportation and Advanced Systems Laboratory, Georgia Tech Research Institute, Georgia Institute of Technology, Atlanta, Georgia, United States of America; 2 Richard B. Russell Research Center, Agricultural Research Service, United States Department of Agriculture, Athens, Georgia, United States of America; University of Illinois at Chicago, UNITED STATES

## Abstract

In nature, protozoa play a major role in controlling bacterial populations. This paper proposes a microfluidic device for the study of protozoa behaviors change due to their chemotactic response in the presence of bacterial cells. A three-channel microfluidic device was designed using a nitrocellulose membrane into which channels were cut using a laser cutter. The membrane was sandwiched between two glass slides; a *Euglena* suspension was then allowed to flow through the central channel. The two side channels were filled with either, 0.1% peptone as a negative control, or a *Listeria* suspension respectively. The membrane design prevented direct interaction but allowed *Euglena* cells to detect *Listeria* cells as secretions diffused through the nitrocellulose membrane. A significant number of *Euglena* cells migrated toward the chambers near the bacterial cells, indicating a positive chemotactic response of *Euglena* toward chemical cues released from *Listeria* cells. Filtrates collected from *Listeria* suspension with a series of molecular weight cutoffs (3k, 10k and 100k) were examined in *Euglena* chemotaxis tests. *Euglena* cells were attracted to all filtrates collected from the membrane filtration with different molecular weight cutoffs, suggesting small molecules from *Listeria* might be the chemical cues to attract protozoa. Headspace volatile organic compounds (VOC) released from *Listeria* were collected, spiked to 0.1% peptone and tested as the chemotactic effectors. It was discovered that the *Euglena* cells responded quickly to *Listeria* VOCs including decanal, 3,5- dimethylbenzaldehyde, ethyl acetate, indicating bacterial VOCs were used by *Euglena* to track the location of bacteria.

## Introduction

Protozoa are unicellular eukaryotes that are nearly ubiquitous in various natural and man-made ecosystems including terrestrial, aquatic, abiotic and biotic habitats[1]. They play a major role in controlling populations of bacteria in soil and other natural ecosystems and they are considered to be a major trophic pathway, whereby biomass produced by microorganisms reenters the food web[2]. Phagotrophic grazing can affect the quantity, activity, and physiological state of prey organisms[3]. Indeed, protistan assemblages may graze on the order of 25 to >100% of the measured daily production of prokaryotic plankton, resulting in the consumption of prokaryotic biomass at approximately the same rate as it is produced[4, 5]. Predation has been demonstrated to be an important determinant of bacterial survival in the environment, accounting for up to 90% of bacterial mortality in aquatic habitats[6].

There is a good deal of evidence suggesting that planktonic protozoa are capable of selective feeding although their feeding preferences are not well understood[7]. A release of dissolved chemical cues, prey motility, prey biochemical composition or nutrient stoichiometry, cell surface characteristics, and prey size might potentially influence selective feeding[8]. Preferential feeding has been reported for protozoans in soil ecosystems with bacterial genera such as *Pseudomonas* being favored over other genera such as *Streptomyces* and *Bacillus[9]*. Jezbera *et al*. observed the preferable predation by the flagellates *Bodo saltans* and *Goniomonas sp*. on *Aeromonas hydrophila* over *Pseudomonas fluorescens[10]*. One recent study suggested that protozoa preferentially graze non-viable and particle-associated forms over viable and free cells[11]. Another paper indicated that *Tetrahymena* sp. tended to preferentially remove the more dominant bacterial species from the community while *Poterioochromonas* sp. and *Acanthamoeba* sp. grazed non-specifically[12]. It appears that protistan species use diverse selection mechanisms to differentially ingest and metabolize bacteria[13].

In turn, bacteria have adapted multiple strategies to counteract predation by protozoa through both intracellular and extracellular defense strategies[14]. Intracellular adaptations include survival and replication of bacteria inside the protozoan cell. Extracellular avoidance mechanisms include altered cell morphology, increased bacterial motility, biofilm formation and production of bioactive compounds. For example, the pelagic bacterium *Flectobacillus* sp. produces long filamentous cells, which are harder to ingest, in the presence of the bacterivorous flagellate *Ochromonas* sp.[15]. Some protozoan activity may also favor bacterial survival. *Acanthamoeba* spp. and some ciliates possess the ability to package bacterial pathogens including *Legionella pneumophila*, *Salmonella enterica*, *Listeria monocytogenes*, and *Escherichia coli* O157:H7, into multilamellar bodies (MLBs)[16]. Bacteria packaged and expelled in MLBs have greater resistance to unfavorable conditions. It was shown that *L*. *pneumophila* packaged in MLBs by the ciliate *Tetrahymena* was more infectious and displayed greater resistance to gentamicin and longer survival in a nutrient-poor environment, compared to the planktonic state[17]. It has been suggested that some waterborne protozoa might act as vehicles and reservoirs to increase the survival of *Campylobacter* in intensively reared poultry[18].

There appear to be molecular interactions between protozoa and bacteria which may employ dissolved cues for chemical-mediated prey selection. Chemicals involved in chemosensory attraction could include proteins, amino acids, and other dissolved inorganic or organic nutrients[19, 20]. Other prey metabolites may prevent grazing, including pyrrolnitrin, 2, 4-diacetylphloroglucinol, hydrogen cyanide, and pyoluteorin[21, 22]. Experiments with bacterial cell extracts indicated that ciliates use dissolved chemical cues to locate biofilms[23].

Co-culture based methods, incubating a mixture of protozoa and bacteria species in a saline buffer and counting the cell numbers over the incubation period, are typically used to study interactions of protozoa and bacteria[17, 24–27]. These methods are straightforward. Unfortunately, co-culture work is time-consuming and it is difficult to monitor behavior change of protozoa in the presence of bacterial species or to study the interactions of protozoa with multiple bacterial species. A capillary tube filled with bacteria embedded in agar can be used to study the swimming pattern of protozoa[28]. It was discovered that under the attraction of bacterial metabolite, phagotrophic protists are capable of congregating at point sources of food within a few minutes, from distances of up to several cms in the case of ciliates, or several mms in the case of microflagellates. Such studies are complicated by the need to control chemical gradients.

In the current study, we studied the interaction between protozoa and bacteria in a microfluidic device made of a porous nitrocellulose membrane. Microfluidic devices have been developed to generate and maintain a stable chemical gradient for bacterial chemotaxis studies[29–33]. *Euglena gracilis* and *Listeria* were selected as the model protozoa and bacterial organisms respectively; *Euglena gracilis* is a unicellular flagellated photosynthetic alga; the body is approximately 10 μm wide and 50–100 μm long. *E*. *gracilis* is a freshwater flagellate found in many aquatic habitats especially shallow eutrophic ponds[34]. *Euglena* can grow photoautotrophically, photo-heterotropically or heterotropically. It has emerged as an excellent source of dietary protein, pro(vitamins), lipids, and the β-1,3-glucan paramylon[35]. Meanwhile, *Listeria* spp., including two pathogenic species *Listeria monocytogenes* and *Listeria ivanovii*, can thrive in various environments and are often isolated from water, soil and detritus[36]. Given the prevalence of free-living protozoa in food-related environments, it is hypothesized that these organisms play an important yet currently under investigated role in the epidemiology of foodborne pathogenic bacteria[37].

## Materials and methods

### Microfluidic device fabrication

Details of the design and fabrication of the microfluidic device have been described previously[30]. Briefly, a CO_2_ laser cutter (Hermes LS500XL) was used to generate a pattern of channels into a nitrocellulose membrane with a thickness of 120 μm (EMD Millipore). A fabricated membrane was soaked in the sterilized and 0.22 μm filtered 0.1% peptone buffer. The wetted membrane was then sandwiched between two pre-cleaned glass microscope slides that were secured with clips. The top slide contained six holes connected to reservoirs made with glass tubes. These holes aligned with the input and output of the three channels in the membrane respectively. The side channels were filled with a control buffer (0.1% peptone) and a chemoeffector solution respectively by adding 100 μl of each solution into the reservoirs connected with side channels. The liquid flows in the side channels were achieved through a capillary force and reached a static equilibrium before the chemotaxis experiment started. A syringe pump was used to control the flow of liquid in the center channel. The device was then fixed on the stage of an inverted confocal microscope (Nikon Eclipse TI) where the movement of protozoa could be observed and recorded.

### Sample preparation

#### Protozoa

*Euglena* was obtained from Carolina Biological Supply Company (Burlington, NC) and maintained in a pre-made *Euglena* media distributed by Carolina Biological Supply Company. The pre-existing bacterial cells inside the *Euglena* suspension were removed by passing the protozoa suspension through a syringe filter with 5 μm pore size and exchanging *Euglena* growth medium with sterilized 0.1% peptone. The cleaned *Euglena* sample was kept in the dark at 21°C for 2 hours before use. A 4-chip hemocytometer from Bulldog Bio (Portsmouth, NH) was used to quantify *Euglena* cells. A concentration of *Euglena* suspension of ~ 10^6^ cell/ml was employed in the chemotaxis experiment.

#### Polystyrene beads

Polystyrene beads with a diameter of 6.1 μm were obtained from ThermoSci. (Waltham, MA). They were diluted in 0.1% peptone and 100 μL of 10 ppm of polystyrene beads were tested in the microfluidic device as a negative control to examine the effect of liquid flow on the distribution of beads inside the microfluidic channel.

#### Bacterial sample

*Listeria monocytogenes* (ATCC 13932) was obtained from Microbiologics (St. Cloud, Minnesota). All growth media and buffers were autoclaved and filtered through 0.22 μm syringe filters. Cultures of each isolate were incubated for 20 hours at 37°C in Trypticase Soy Broth (TSB, Fisher Scientific, Pittsburgh, PA, USA). Overnight cell cultures were centrifuged three times and pellets were suspended in 0.1% peptone to produce a concentration of 10^9^ cfu/ml. Optical densities (600 nm) of bacterial cultures were adjusted to produce a suspension containing 10^9^ cfu/ml, and suspensions were serially diluted in 0.1% peptone to produce a suspension of the desired concentration. Suspensions of dead bacteria were prepared by autoclaving suspensions of live bacteria at 121°C for 15 minutes.

#### Filtrates collected with different molecular weight cutoffs

Bacterial samples from overnight cultures underwent buffer exchange with a 0.22 μm syringe filter from growth media to 0.1% peptone. Bacteria remained in the 0.1% peptone buffer for approximately 30 minutes to allow any chemicals released from cells to diffuse into the media. The sample was then loaded into an EMD centrifugal filter with molecular weight discriminations of 3kD, 10kD, or 100kD. Filtrates with a molecular weight below these thresholds were collected and used as the experimental attractant in the microfluidic device.

#### *Listeria* VOC spiked samples

Bacterial samples from overnight cultures underwent buffer exchange with a 0.22 μm syringe filter from growth media to 0.1% peptone in an airtight glass vial. A second airtight glass vial containing 10 ml of filtered 0.1% peptone solution was then connected to the vial containing the *Listeria*/peptone via a PTFE tube. The *Listeria* vial was gently heated to around 35°C, and both vials were held for roughly 2 hours to allow for a transfer of headspace VOCs from the *Listeria* vial to the second vial. Afterward, the second vial was detached from the *Listeria* vial and gently inverted.

Four representative bacterial VOCs including 2-ethylhexyl acetate, ethyl acetate, 3,5-dimethylbenzaldehyde, and decanal were selected for further chemotaxis validations. They were purchased (Sigma Aldrich, St. Louis, USA) and used directly. The VOC spiked samples were prepared in 0.1% peptone water individually at their saturation concentrations and 10-fold (100-fold dilution for ethyl acetate) dilution of their saturated solution in 0.1% peptone was used for euglena chemotaxis experiment.

### Experimental design

**Fig 1:**
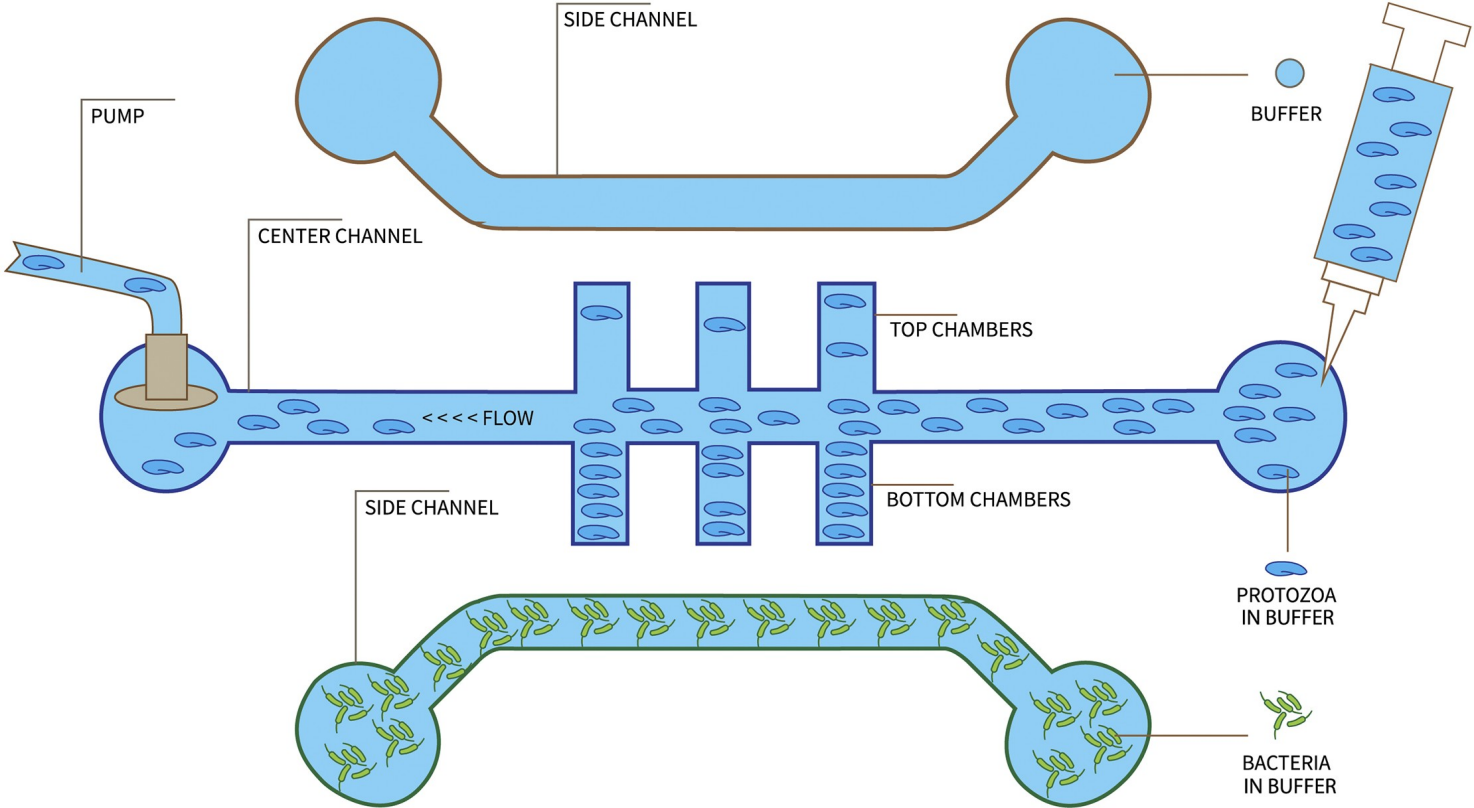
Conceptual design of the microfluidic device used in this study. The sample was introduced from the right end of the center channel. The chambers are named as 1^st^, 2^nd^, and 3^rd^ (from right to left) set of chambers to represent the sequences of a sample to reach the entrances of the chambers.

**Fig 2:**
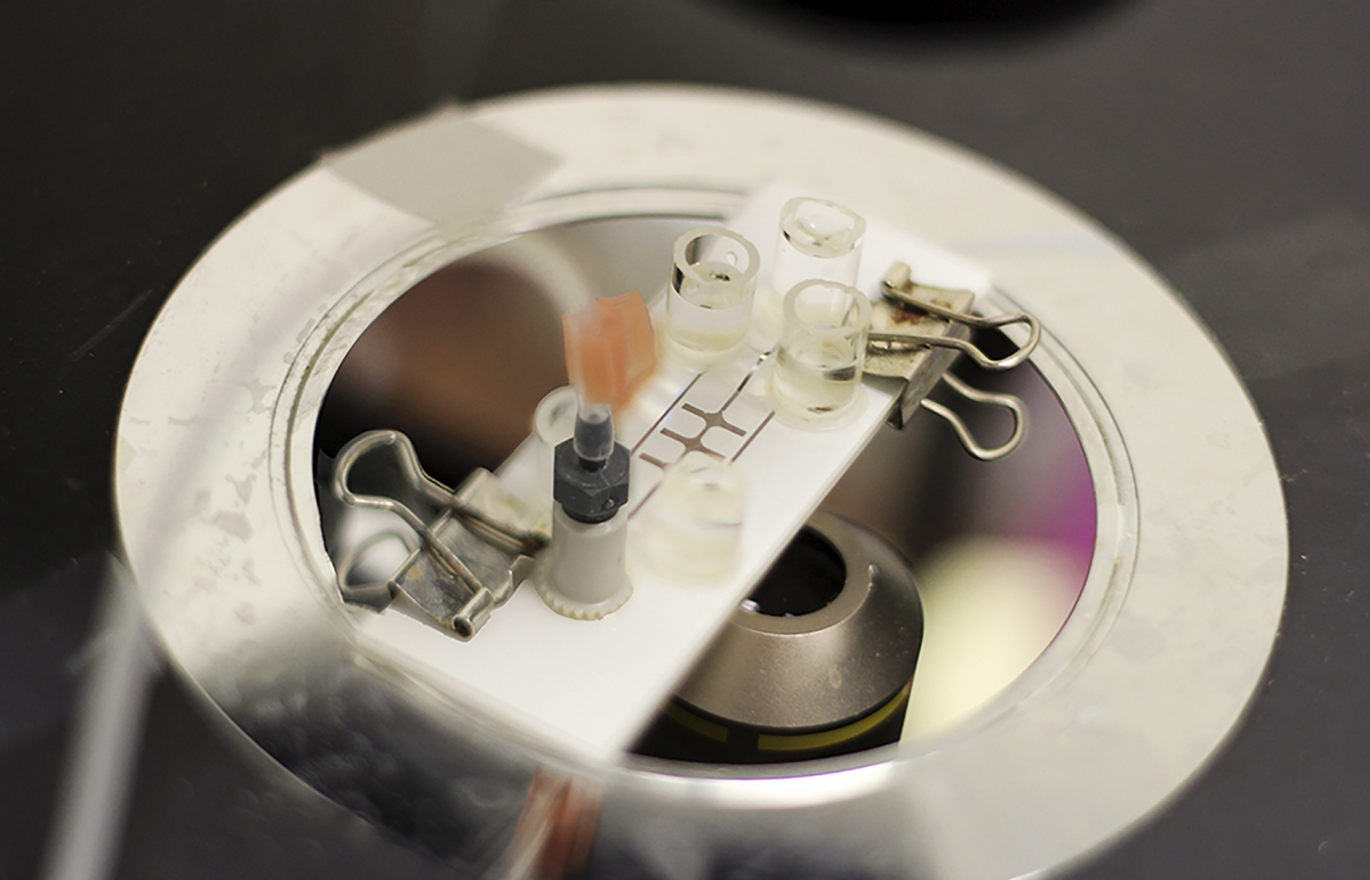
Microfluidic assembly on the stage of the microscope. A syringe pump was used to control the flow rate of the *Euglena* suspension. *The Euglena* sample was added in the reservoir on the right end of the central channel and pulled through the microfluidic system using a syringe pump connected on the left.

Our microfluidic design contains a center channel and two side channels, as shown in Fig 1. The center channel includes six dead-end side chambers, three pointing towards the top side channel and three toward the bottom. The *Euglena* suspension was added to the center channel and the flow rate was controlled by a syringe pump, as shown in Fig 2. The side channels were filled with chemoeffectors and control buffer respectively.

Channels in assembled microfluidic devices were washed and filled with 0.1% peptone using a vacuum pump. A volume of 100 to 200 μL *Euglena* suspension was loaded into the center channel at a flow rate controlled by a syringe pump. The flow rate of the *Euglena* suspension was set as 3 μL/h. Both of the side channels were filled with 0.1% peptone. After allowing the system to equilibrate with *Euglena* in the microfluidic channel for 20 minutes, the cell counts process was started and designated at time 0, which was right before the introduction of chemoeffector in the side channel. At 20 minutes, 100 μL of the bacterial cell suspension or filtrate solution collected from bacterial samples were added into the bottom channel of the device. Videos were then taken at 10-minute intervals until 60 minutes to determine if a change in *Euglena* cell distribution had taken place. These experiments were performed in a dark room to prevent *Euglena* from responding to ambient light instead of the chemoeffector being tested.

### Data collection and analysis

Videos were taken of the three perpendicular chambers of the device as identified in Fig 1 to analyze any change in distribution towards channels containing chemoeffector compared to channels containing just buffer. After each video was recorded, data was taken from the videos with Nikon NIS Elements. A screenshot of the video was prepared for *Euglena* cell counts. The viewing area of the chamber was measured and kept standard for all measurements. *Euglena* cells were counted using Nikon Elements software. After counting *Euglena* at the top and bottom of each channel, the percentage of a cell population in the top and bottom of each channel was compared at each time point. The total number of *Euglena* (the sum of cell numbers in the top and bottom chambers) at each time point was used to calculate the percentage of cells at the top and bottom chambers. If *Euglena* had a preference for bacteria or other attractant placed in a channel then the percentage of *Euglena* near that channel should increase over time to above 50% and stay above 50%. Each test was repeated at least three times and the mean value with standard deviation are reported.

### VOC identification

A 10 mL aliquot of bacterial VOC sample was transferred to a 20 mL capped glass vial. The vial was heated at 50°C and stirred at 200 rpm for 20 min to equilibrate liquid and vapor, then the organic vapors in headspace were adsorbed on a SPME fiber coated with 50/30 mm DVB/CAR/PDMS (Gerstel, Linthium, MD, USA) for 20 min at 50°C. The sample was desorbed at 250°C for 2 min in the GC injector in splitless mode. The VOC analysis was performed using a Pegasus 4D GC×GC-TOFMS instrument (LECO, St. Joseph, MI, USA), including an Agilent 6890 GC (Agilent Technologies, Palo Alto, CA, USA), time of flight mass spectrometer (LECO) and Gerstel MPS2 autosampler (Gerstel, Mülheim, Germany). A 30 m × 0.25 mm id. × 0.25 μm d_f_ Rxi-17sil column (Restek Corp., Bellefonte, PA) was connected to a 2 m × 0.25 mm i.d. × 0.25 μm DB5 column (Restek Corp., Bellefonte, PA) in series and separated by the cryogenic modulator so that the polar primary column separated VOCs according to their polarity and the non-polar secondary column in the secondary oven separated VOCs according to their boiling point. VOCs eluting from the secondary column were detected with TOF-MS. Helium was used as the carrier gas at 2.0 mL/min controlled via an automated pressure ramp. An initial temperature of 45°C held for 1 min, followed by a gradient at 5°C/min to 120°C, then 10°C/min to 240°C was used in all analyses. The second column was operated at 5°C higher than the primary column. The transfer line was at 240°C. The thermal modulator offset was +15°C relative to the primary oven temperature. The modulation period was 5 seconds. The MS range of mass-to-charge ratio (m/z) was 35–450 and 200 mass spectra were acquired per second. The ion source chamber was held at 200°C. The detector voltage was 15750 V with an electron energy of 70 eV.

Alignment on chromatographic data was conducted using the Statistical compare feature of the ChromaTOF^®^ software, v4.50 (LECO). The baseline was obtained between the middle and the top of the noise. Peaks with a minimum signal to noise ratio of 100 were identified. Subpeaks were combined if shifts on their second dimension retention time were less than 0.1 s and their mass spectral match was greater than 600/1000. Identified peaks were assigned putative identifications based on mass spectral matching score greater than 700/1000 compared to the National Institute of Standards and Technology (NIST) 2011 mass spectral library. All post GC-MS data analysis including clustering analysis and feature identification were performed with Python programs using routines from the sklearn and scipy libraries[38].

## Results and discussion

Using rapid prototype techniques including a laser cutter based method to write patterns directly on the nitrocellulose membrane provides a simple and maskless fabrication process to produce microfluidic devices. The process is simple and the microfluidic design can be easily modified and adapted in the process of laser writing. We have developed a design containing chambers perpendicular to the center channel so the movement of studying microorganisms inside the chambers will not be influenced by the variations in the fluid flow. Our previous study indicated that a pseudo-linear chemical gradient inside the chamber can be established within 10 minutes and maintained for at least 60 minutes via a chemical diffusion through the porous nitrocellulose membrane[30]. Using porous membranes to generate a stable static chemical gradient for bacterial chemotaxis study has also been reported by Diao et al[39]. Other porous media including porous trap fabricated from the protein bovine serum albumin (BSA) by a dynamic mask-based multiphoton lithography (MPL) [40], poly(2-hydroxyethyl methacrylate-co-ethylene dimethacrylate) (HEMA–EDMA) based hydrogel[41], and polycarbonate membrane[42] have been reported to study bacterial chemotaxis. Porous media are permeable to nutrients, waste products, and other bioactive small molecules but confine the microorganisms in the defined locations. In addition, the static flow-free chemical gradient based on the porous membrane is more suitable for chemotaxis studies as the challenges associated with flow-based gradient generators including short residence time, shear flow impact on the movement of microorganisms are greatly suppressed[43].

**Fig 3:**
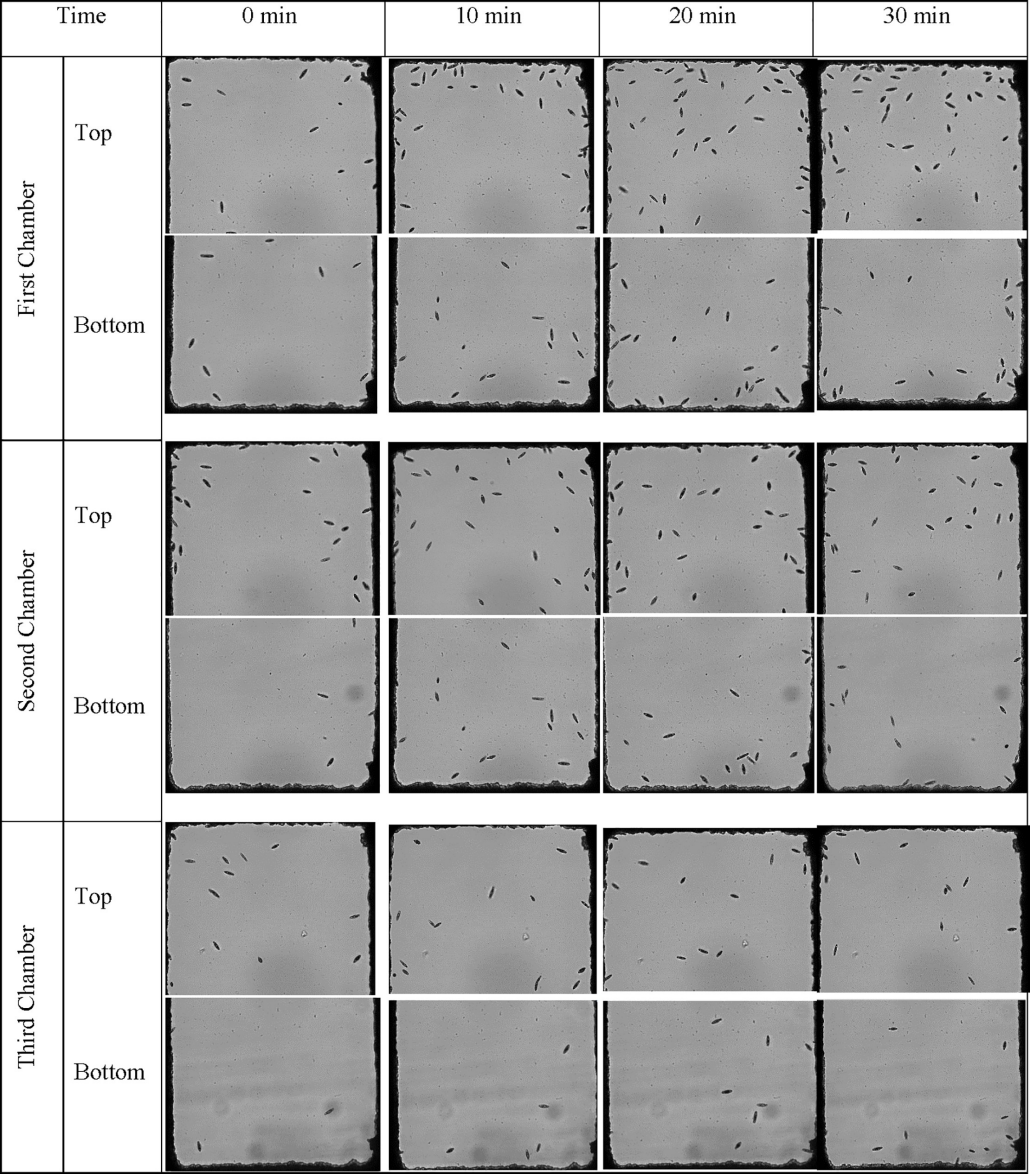
*Euglena* cell distribution inside the microfluidic device with two side channel filled with 0.1% peptone during the experimental duration. The flow rate of *Euglena* suspension was 3 μL/h. Pictures were taken at the end of each set of chambers at 0 min, 10 min, 20 min, and 30 mins after the chemmoeffector (0.1% peptone) was introduced in the microfluidic system.

The behavior of *Euglena* inside the microfluidic devices was studied first with both side channels filled with sterilized 0.1% peptone. The flow rate of the *Euglena* suspension was set as 3 μL/h. Faster flow rates at 15 and 30 μL/h were also examined, however, *Euglena* cells were pulled through the device too quickly to allow for a chemotactic shift. In addition, the high shear rate caused by a faster flow produced heterogeneous cell distributions in the form of regions of accumulation and regions of depletion[44]. Based on the current microfluidic design, the volume of the center channel is about 0.3 μL so the residence time for a *Euglena* cell is about 6 minutes. Although there are no chemoeffectors inside the side channels, more *Euglena* cells reached the topsides of the chambers after they were introduced inside the microfluidic channel for 20 minutes. Chamber 1 (the chamber nearest sample introduction) contained more *Euglena* cells than the chambers closer to the exit of the central channel as shown in Fig 3.

**Fig 4:**
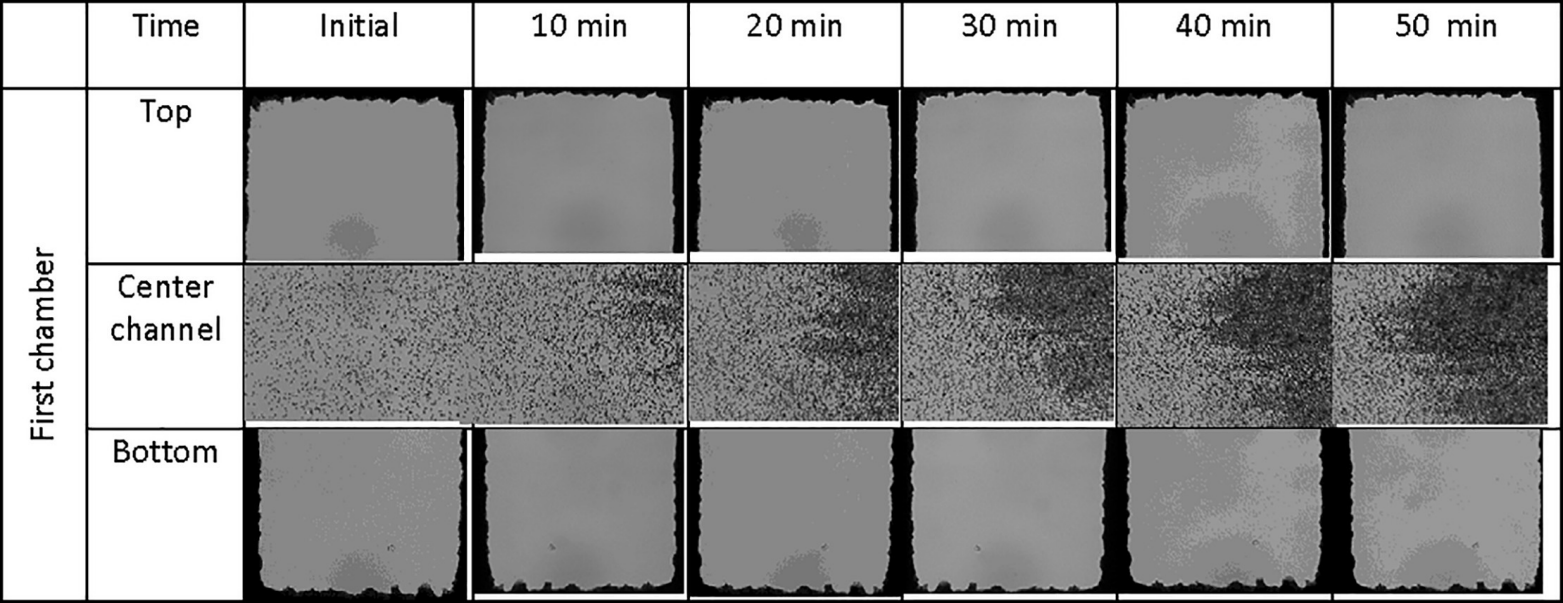
A suspension of 6 μm polystyrene beads was pulled into the center channel at a flow rate of 3 μl/h by a syringe pump. The pictures were taken at the both ends of the first chamber and parts of center channel between the entrances of the first set of chambers. It was observed that the beads did not reach the bottom of chambers and they had a tendency to accumulate in the central channel.

Forward swimming of *Euglena* cells occurred at a rate of 13.7 μm/sec at 6°C and increased to a maximum of 80.1 μm/sec at 30°C[45]. The swimming rate of *Euglena* was about 50 μm/sec at room temperature. Therefore, it took less than 132 sec for *Euglena* cells to cross the end of the top chamber to the end of the bottom chamber based on our current design. It was observed that the number of *Euglena* cells inside chambers gradually increased over time and slightly more *Euglena* cells were found in the top chambers, as shown in Fig 3. As a comparison, the suspension of 6 μm polystyrene beads, used as a non-motile control, did not reach the bottom of the chambers due to the lack of motility. It was observed that more beads accumulated in the center channel directly between the entrances of the first set of the chambers and concentration decreased towards the third set of the chamber, as shown in Fig 4. This could explain why a higher concentration of *Euglena* is observed in the first channel versus the other two channels.

**Fig 5:**
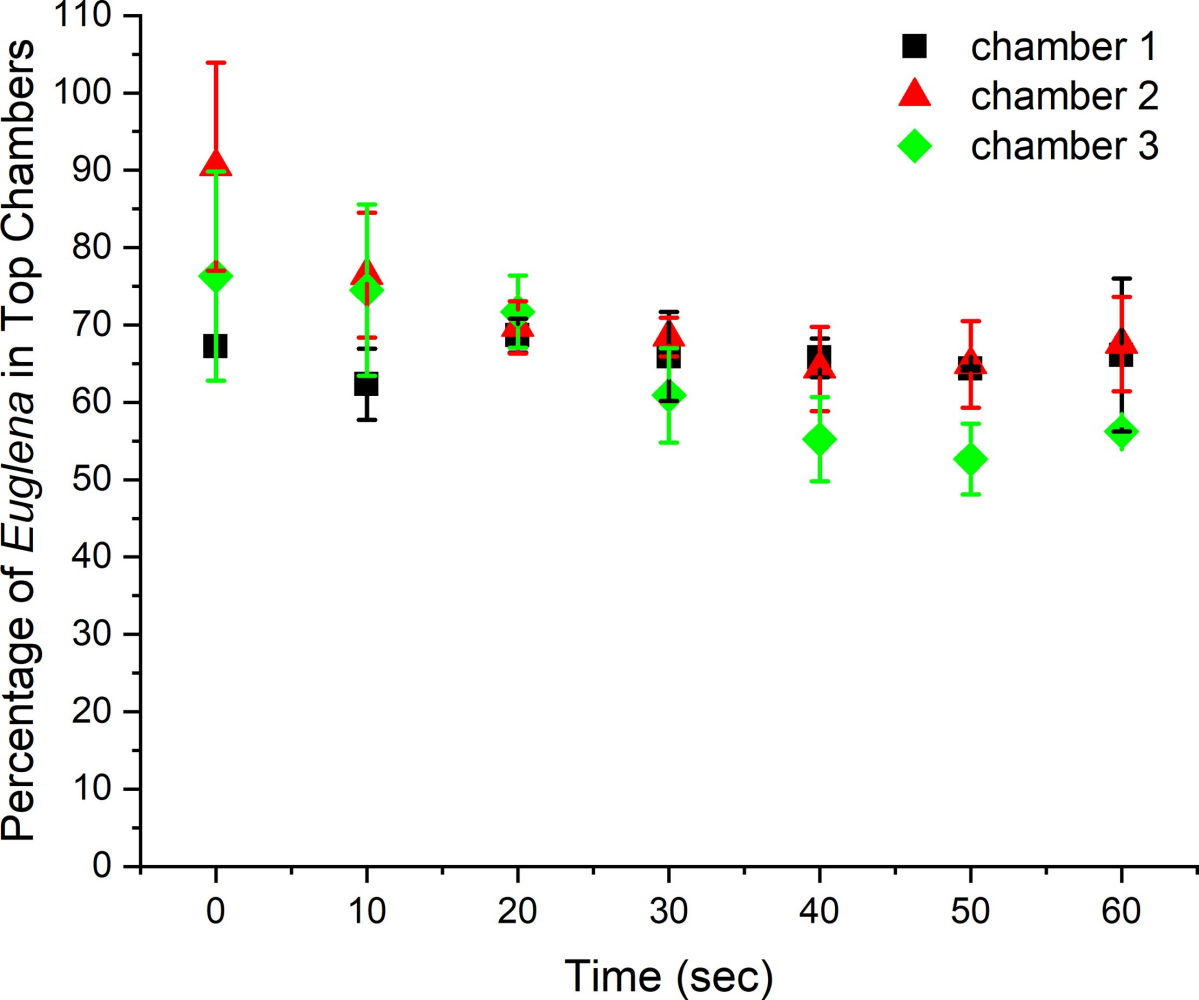
*Euglena* distribution (percentage of observed cells in top chambers over time) inside the microfluidic chambers without a chemoeffector. Uneven distribution of the illumination light from the microscope caused more cells to move to the top of the chambers.

**Fig 6:**
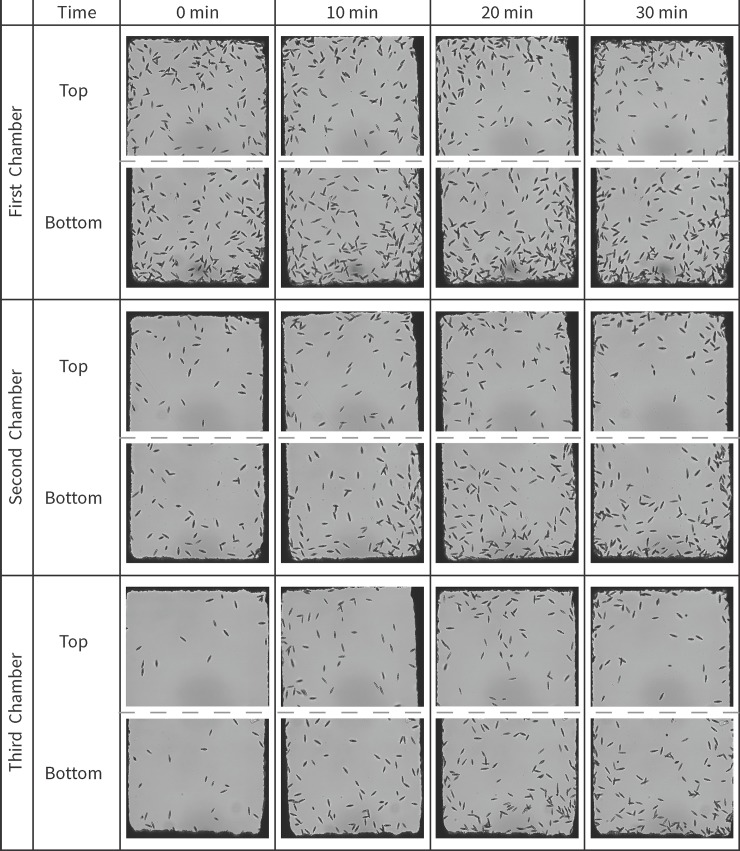
***Euglena* cell distribution inside the microfluidic device with one side channel (top) filled with 0.1% peptone and the other side channel (bottom) filled with *Listeria* suspension during the experimental duration.** The flow rate of *Euglena* suspension was 3 μL/h. Pictures were taken at the both ends of the chambers and used for cell distribution analysis.

**Fig 7:**
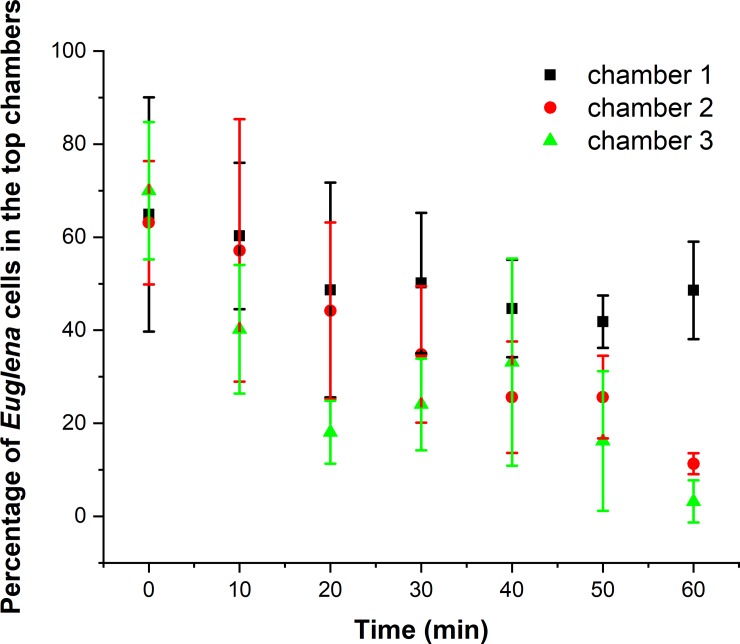
*Euglena* distribution (percent of total observed cells over time from initiation of experiment) inside the microfluidic chambers with the bottom side channel filled with *Listeria* suspension.

Slightly more *Euglena* cells shifted to the chambers on the top even when both side channels were filled with 0.1% peptone. This observation could be attributed to the uneven light distribution on the microscope stage as the *Euglena* is photosensitive [46, 47]. Although the experiment was conducted in a light-tight room, the illumination light from the microscope was on for roughly 5 minutes during the video collection period and potentially affected the motion. The cell distribution in the three chambers was relatively stable during the duration of the experiment. Greater than 50% of *Euglena* cells (about 65%) remained in the top chambers during the entire experimental duration, indicating a slightly biased *Euglena* accumulation in the top chambers, as shown in Fig 5. To avoid compounding effects from the light, the chemoeffectors either attractants or repellent[30], were introduced to the side channel closest to the bottom of the chambers. The tested chemoeffectors included bacterial suspension and filtrates collected with different MW,

*Listeria* cell suspension in 0.1% peptone was added into the side channel close to the bottom chambers and the top channel was filled with 0.1% peptone as the control. The shift of *Euglena* cells inside the microfluidic chambers over time can be found in Fig 6. At time 0 min, more than 60% of the *Euglena* in the viewing section of each chamber were in the top of the chamber, as expected from the previous experiment. The percentages of *Euglena* cells in the top chambers were quickly reduced after *Listeria* was introduced into the system. It’s interesting to observe that the reductions in percentages of *Euglena* cells in the top of chamber 2 and 3 were similar while less reduction was observed in chamber 1. The rates of reduction in 1^st^, 2^nd^, and 3^rd^ chambers, by linear fitting the data in Fig 7, are 0.3%/min, 0.86%/min, and 0.67%/min respectively. The significant population shifts of *Euglena* inside the chambers suggests that chemicals released from *Listeria* attracted the protozoa. The pore size of the nitrocellulose membrane was about 0.22 μm, which is small enough to prevent *Listeria* cells from entering chambers housing *Euglena*, while allowing molecules released by *Listeria* to reach the chambers. The absolute number of *Euglena* cells inside the first chambers are much higher compared to those in the second and third chambers. The higher cell density might result in an increased cell dispersion inside the chambers due to the abiotic stress associated with higher cell densities [48], which might have a negative impact on the chemotactic behavior of *Euglena*. The chemotactic response of *Euglena* to other bacterial cells including *E*. *coli*, *P*. *aeruginosa*, and *S*. *aureus* were also examined inside the microfluidic device. Similar population shifts of *Euglena* cells were observed indicating the response of *Euglena* towards *Listeria* might not be selective.

To narrow down the chemical cues responsible for attracting *Euglena*, we used filtrates collected from filters with different molecular weight cutoffs including 3k, 10k and 100k. The filtrate was then introduced into the side channel instead of the whole cell suspensions. We observed similar *Euglena* distribution inside the microfluidic devices even with the filtrates collected using the 3k MW cutoff membrane, indicating that chemicals with molecular weight less than 3k could be attractants for *Euglena*. To further identify the chemical cues responsible for the population shift of *Euglena* cells, headspace VOCs released from *Listeria* cells were collected in 0.1% peptone water and used as chemoeffectors in the side channel. The percentages of cells in the top chambers were reduced from 65% to ~ 25% after *Listeria* VOC was introduced in the side channel next to the bottom chambers for 60 min, suggesting *Listeria* VOCs might be the chemical cues for *Euglena*. It is well known that bacteria release molecules of low molecular weight (<300 Da) and high vapor pressure (0.01 kPa at 20°C) that can readily evaporate and diffuse through heterogeneous mixtures of solids, liquids and gasses[49]. Volatiles were known to assist cross-kingdom interactions, such as plant–insect communications as bi- and tritrophic attractions and defenses[50–53].

**Fig 8:**
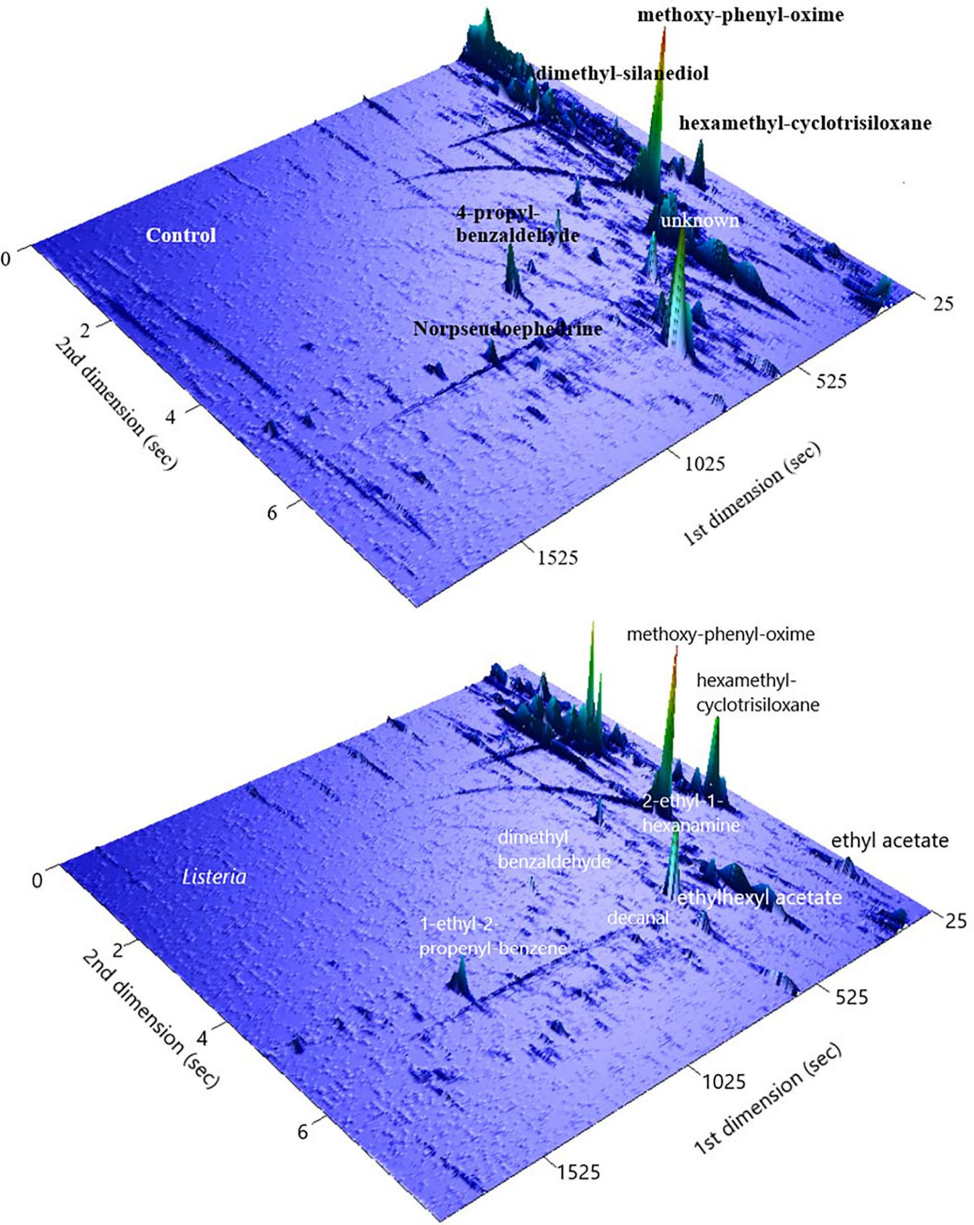
**Total ion GCXGC chromatographs of a peptone buffer control (top) and a *Listeria* VOC sample (bottom) showed different VOC features present in VOC sample compared to the buffer.** Peaks can be located with two retention times as x and y axis and peak intensity is in z axis.

Headspace SPME-GCxGC-TOFMS was used to identify *Listeria* VOCs present in 0.1% peptone buffer. Both headspace samples from *Listeria* VOC spiked 0.1% peptone and 0.1% peptone buffer were examined and compared. Over two hundred VOC compounds were identified in both control (0.1% peptone only) and samples (VOCs from *Listeria* diffused into 0.1% peptone). Typical 2-dimensional GC plots with retention times from two columns and corresponding peak intensities can be found in Fig 8. Some of the identified VOCs including siloxanes from the SPME fiber were also labeled in the chromatograms.

**Fig 9:**
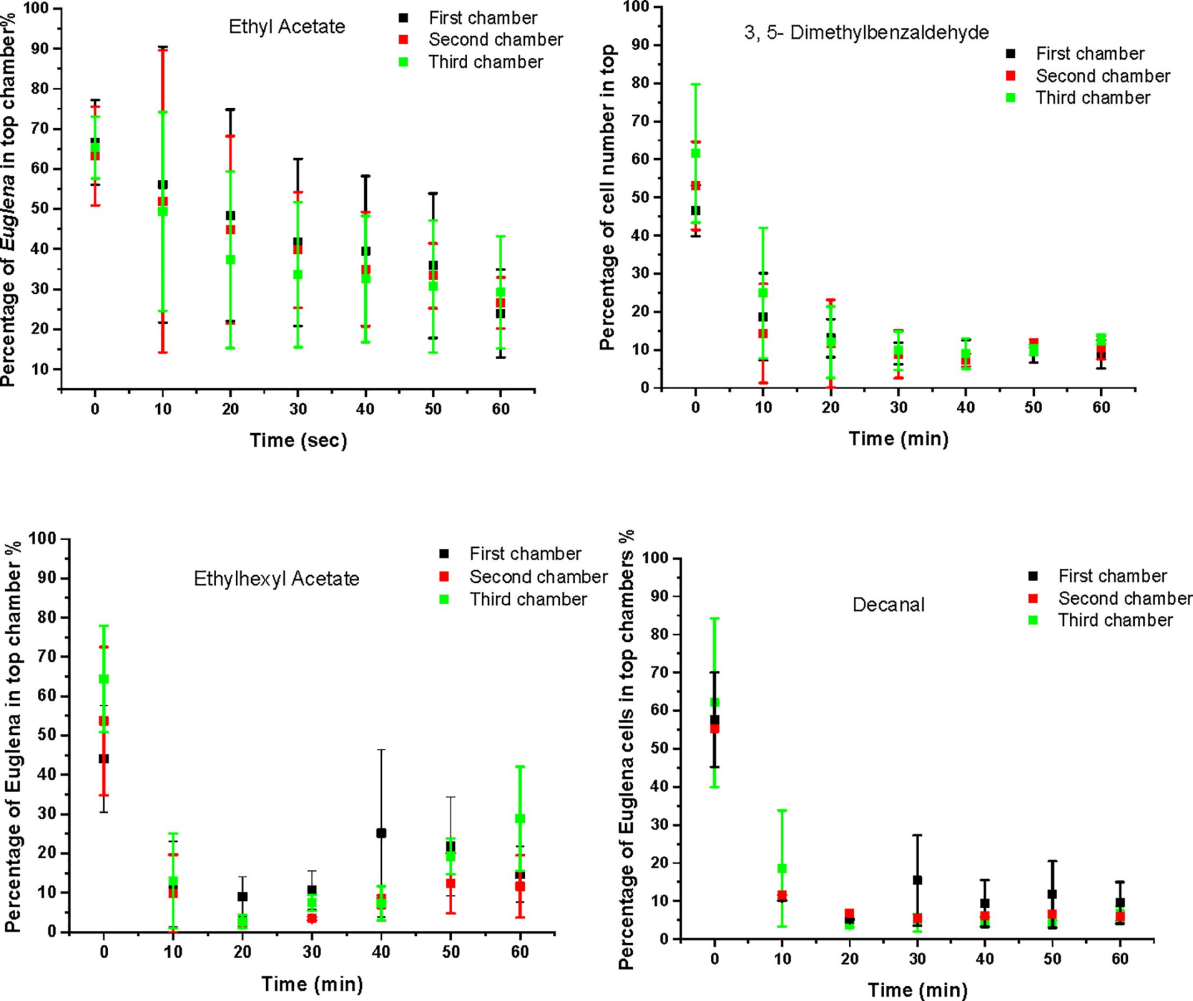
Population shift of *Euglena* cells inside the microfluidic system with different VOC molecules served as chemoattractant. Significant population shift can be observed when ethylhexyl acetate and decanal were used as chemoattractants.

**Fig 10:**
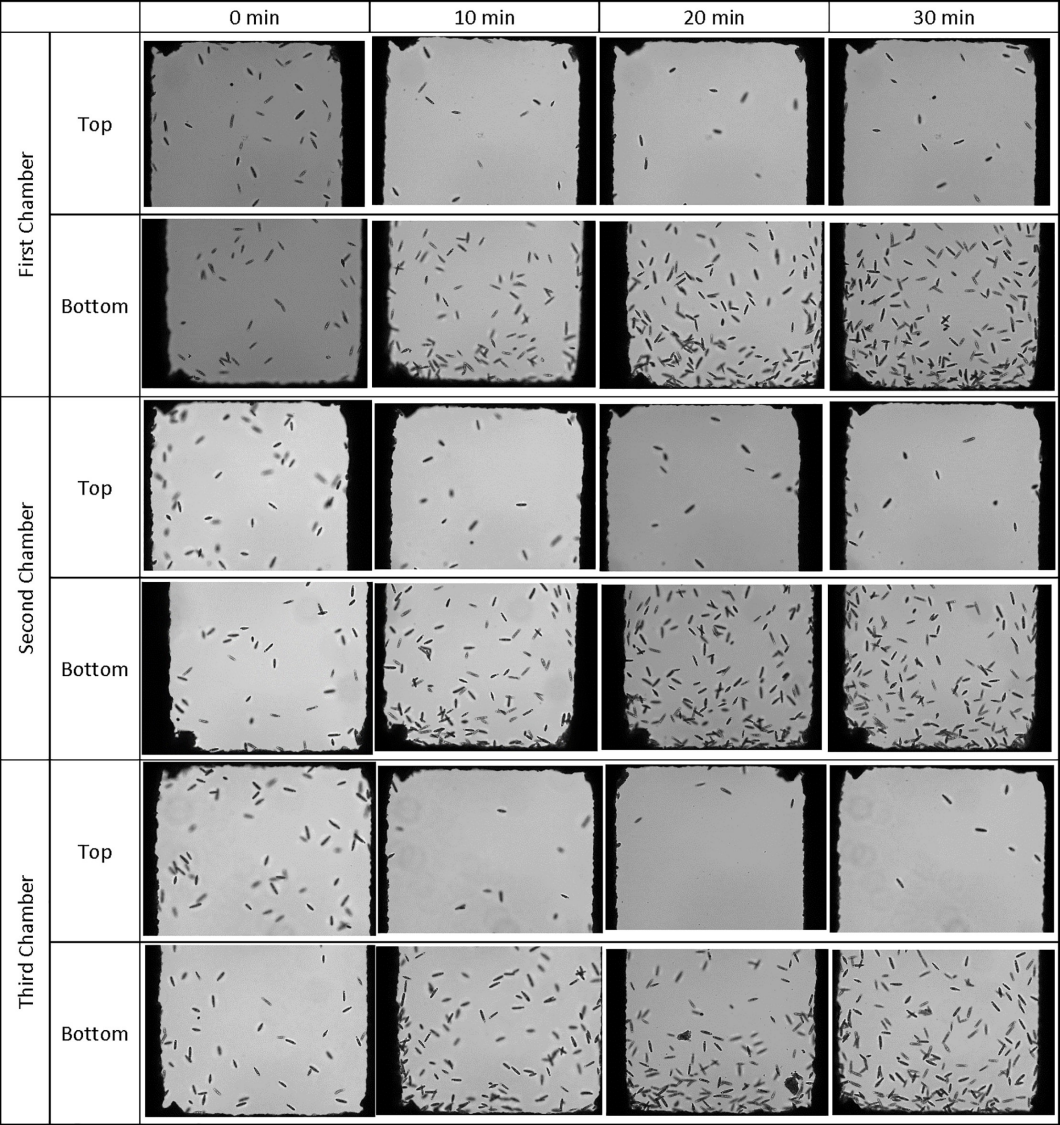
***Euglena* cell distribution inside the microfluidic device with one side channel (top) filled with 0.1% peptone and the other side channel (bottom) filled with decanal spiked 0.1% peptone during the experimental duration.** The flow rate of euglena suspension was 3 μL/h.

A Random Forest based algorithm was used to identify the most significant VOCs from *Listeria* GC-MS datasets comparing to the datasets to the background[54]. The algorithm constructs random decision trees, fits them to the dataset, and uses their averaged output for classification or regression. VOCs that were either only present in the bacterial VOC samples or have much higher levels compared to the controls were extracted from the Random Forest analysis. Those compounds, are grouped into four categories including esters, aldehyde, alkanes, and others listed in Table 1. Four representative VOCs including 2-ethylhexyl acetate, ethyl acetate, 3,5-dimethylbenzaldehyde, and decanal were selected for further chemotaxis validations as they showed higher significances and have been reported as bacterial VOCs in the literature[55, 56]. They were spiked in 0.1% peptone and used as chemoeffectors inside the microfluidic device. Significant shifts on *Euglena* cells were observed towards all four tested VOCs. The rates of changes (the slope of curve fitting of plots in Fig 9 from 0 to 20 min) were used to compare the significance of each VOC as an attractant for *Euglena*. The data can be found in Table 2. Decanal was shown to be the most significant attractant because the presence of small amounts of decanal (lowest solubility) can introduce the *Euglena* to shift at a speed of 2.5%/min. As a comparison, the shift of *Euglena* over time with decanal as the chemoeffectors can be found in Fig 10. Most of the *Euglena* shifts were accomplished in 10 minutes.

**Table 1:** Major VOCs produced from *Listeria*.

Type	Chemical	Retention Time		Solubility in water (g/L) at 25°C
		1^st^ dimension (sec)	2^nd^ dimension (sec)	
Ester	2-Ethylhexyl acetate	585	6.35	0.0039
	Pentyl propionate	705	6.64	0.81
	Ethyl acetate	81	2.07	83
	1,2-Benzenedicarboxylic acid bis(2-methylpropyl) ester	1625	4.89	NA
Aldehyde	3,5-Dimethylbenzaldehyde	849	4.38	1.39
	Decanal	689	6.05	1.56e-6
	2-Methylundecanal	473	5.86	0.0036
	Octanal	409	5.23	0.21
Alkanes	1-Ethyl-2,4-dimethylbenzene	481	5.57	0.015
	1,3,5-trimethyl-2-octadecylbenzene	281	4.12	NA
	Cis-bicyclo[4.2.0]octane	553	5.58	NA
Other	1, 3-dichlorobenzene	473	4.75	0.125
	Isopropylsulfonyl chloride	81	5.65	22.4
	Boric acid	265	1.85	50
	2-ethyl-1-hexanamine	553	5.99	1.6

**Table 2:** Rate of change for *Euglena* with each attractant.

Attractant	Concentration	First chamber (%/min)	Second chamber (%/min)	Third chamber (%/min)
*Listeria* cell	~ 10^8^ cfu/ml	0.30	0.86	0.67
Ethyl acetate	~ 0.83 mg/ml	0.68	0.57	0.66
Dimethylbenzaldehyde	~ 0.14 mg/ml	1.64	2.07	2.29
Ethylhexyl acetate	~ 0.0039 mg/ml	1.42	1.71	2.60
Decanal	~ 1.56x10^-6^ mg/ml	2.62	2.42	2.43

Decanal has been reported as a bacteria VOC associated with *Acinetobacter baumannii*[57], *Lysobacter antibioticus*, *L*. *capsici*, *L*. *enzymogenes*[58], lactic acid bacteria (LAB) strains (*Lactobacillus sanfranciscensis*, *Leuconostoc citreum* and *Weissella cibaria*)[59], 25 strains out of 50 bacteria strains in a systematic bacteria VOC study[60], bacteria strains with strong nematicidal activities[61]. In fact, the nematicidal activity of decanal has been shown 100% effective towards free-living nematode *Panagrellus redivivus* and the pinewood nematode *Bursaphelenchus xylophilus*[61]. Decanal is involved in bacterial fatty acid metabolism[62], which might be a biomarker for protozoa to sense the presence of bacteria.

In earlier work on the attraction of protists towards bacteria, a heat-stable chemoattractant was isolated from bacterial cultures. This component had a molecular weight in the range of 500–1000 daltons, was produced by both Gram-positive and Gram-negative bacteria, and served equally well as an attractant for both the bacterial feeding *Paramecium* and for its natural predator, *Didinium[63]*. *Naegleria fowleri* amoebae demonstrated a chemotactic and chemokinetic response toward live cells and extracts of *Escherichia coli* and other bacterial species when experiments were performed using a blind-well chemotaxis chamber. It was also reported that both free-swimming filter feeder *Tetrahymena* sp. and the surface-associated predator *Chilodonella* sp., used dissolved chemical cues from bacterial cell extracts for preferential feeding decisions although the nature of the dissolved cues which caused the feeding preference is uncertain[23]. A recent study on the chemotactic behavior of *Trypanosoma brucei* towards *E*. *coli* indicated that the attractant is diffusible through the culture medium, and produced by actively growing bacteria. However, efforts to isolate the attractant was unsuccessful[64]. Our study suggested that dissolved VOCs released from bacteria cells are used as chemical cues by protozoa to identify and locate the targets. In our knowledge, our report is the first study to show the bacterial VOCs are the chemical cues for protozoa in a liquid phase.

## Conclusions

We have designed and demonstrated the effectiveness of a microfluidic device for the quantitative evaluation of protozoa response to *Listeria monocytogenes*. Chemicals released by bacteria diffuse through the nitrocellulose membrane and act as attractants to the protozoa. The design of this device allows for quantifiable comparisons between the numbers of *Euglena* that move toward either side of the device. The two channels above and below the center channel allow for two different bacteria to be compared or one bacteria against a control. An observable shift of protozoa in the direction of a species bacteria over a control indicates that protozoa are attracted to that bacteria. Comparing the attraction of protozoa towards two different species of bacteria may indicate if the protozoa have a preference towards ingesting certain species of bacteria over others.

We have discovered that *Listeria* VOCs dissolved in a buffer are the chemical cues for *Euglena*. Several VOCs associated with bacterial samples were identified including 2-ethylhexyl acetate, ethyl acetate, 3,5-dimethylbenzaldehyde, and decanal. Their chemoattractant activities toward *Euglena* were confirmed. Among the four VOCs studies, decanal showed the most significant influence to attract *Euglena*. In the future, we can assess an organism’s tendency to make these VOCs, and use that as a measure of how useful protozoa predation could be in a control effort. We may be able to search for the genes that control VOC release and search for those in potential prey using a targeted molecular method.
